# Non-linear dose response of DNA double strand breaks in response to chronic low dose radiation in individuals from high level natural radiation areas of Kerala coast

**DOI:** 10.1186/s41021-023-00273-6

**Published:** 2023-05-01

**Authors:** Vinay Jain, Divyalakshmi Saini, D. C. Soren, V. Anil Kumar, P. R. Vivek Kumar, P. K. M. Koya, G. Jaikrishan, Birajalaxmi Das

**Affiliations:** 1 Low Level Radiation Research Section (LLRRS), Radiation Biology & Health Sciences Division (RB&HSD), Bio-Sciences Group (BSG), Bhabha Atomic Research Centre (BARC), Trombay, Mumbai, 400 085 India; 2 Low Level Radiation Research Laboratory, LLRRS, RB&HSD, BSG, BARC, IRE Campus, Beach Road, Kollam, Kerala 691 001 India; 3 Homi Bhabha National Institute (HBNI), Anushakti Nagar, Trombay, Mumbai, 400 094 India

**Keywords:** Human peripheral blood mononuclear cells, DNA double strand breaks (DSBs), γH2AX foci, Chronic low dose radiation, Normal level natural radiation areas (NLNRA), High level natural radiation areas (HLNRA)

## Abstract

**Background:**

The human population living in high level natural radiation areas (HLNRAs) of Kerala coast provide unique opportunities to study the biological effects of low dose and low dose rate ionizing radiation below 100 mGy. The level of radiation in this area varies from < 1.0 to 45 mGy/year. The areas with ≤ 1.50 mGy/year are considered as normal level natural radiation areas (NLNRA) and > 1.50 mGy/year, as high level natural radiation areas (HLNRA). The present study evaluated dose response relationship between DNA double strand breaks (DSBs) and background radiation dose in individuals residing in Kerala coast. Venous blood samples were collected from 200 individuals belonging to NLNRA (n = 50) and four dose groups of HLNRA; 1.51-5.0 mGy/year (n = 50), 5.01-10.0 mGy/year (n = 30), 10.01-15.0 mGy/year (n = 33), > 15.0 mGy/year (n = 37) with written informed consent. The mean dose of NLNRA and four HLNRA dose groups studied are 1.21 ± 0.21 (range: 0.57–1.49), 3.02 ± 0.95 (range: 1.57–4.93), 7.43 ± 1.48 (range: 5.01–9.75), 12.22 ± 1.47 (range: 10.21–14.99), 21.64 ± 6.28 (range: 15.26–39.88) mGy/year, respectively. DNA DSBs were quantified using γH2AX as a marker, where foci were counted per cell using fluorescence microscopy.

**Results:**

Our results revealed that the frequency of γH2AX foci per cell was 0.090 ± 0.051 and 0.096 ± 0.051, respectively in NLNRA and HLNRA individuals, which were not significantly different (t_198_ = 0.33; P = 0.739). The frequency of γH2AX foci was observed to be 0.090 ± 0.051, 0.096 ± 0.051, 0.076 ± 0.036, 0.087 ± 0.042, 0.108 ± 0.046 per cell, respectively in different dose groups of ≤ 1.50, 1.51-5.0, 5.01-10.0, 10.01-15.0, > 15.0mGy/year (ANOVA, F_4,195_ = 2.18, P = 0.072) and suggested non-linearity in dose response. The frequency of γH2AX foci was observed to be 0.098 ± 0.042, 0.078 ± 0.037, 0.084 ± 0.042, 0.099 ± 0.058, 0.097 ± 0.06 and 0.114 ± 0.033 per cell in the age groups of ≤ 29, 30–34, 35–39, 40–44, 45–49 and ≥ 50 years, respectively (ANOVA, F_5,194_ = 2.17, P = 0.059), which suggested marginal influence of age on the baseline of DSBs. Personal habits such as smoking (No v/s Yes: 0.092 ± 0.047 v/s 0.093 ± 0.048, t_198_ = 0.13; P = 0.895) and drinking alcohol (No v/s Yes: 0.096 ± 0.052 v/s 0.091 ± 0.045, t_198_ = 0.62; P = 0.538) did not show any influence on DSBs in the population.

**Conclusion:**

The present study did not show any increase in DSBs in different dose groups of HLNRA compared to NLNRA, however, it suggested a non-linear dose response between DNA DSBs and chronic low dose radiation.

**Supplementary Information:**

The online version contains supplementary material available at 10.1186/s41021-023-00273-6.

## Introduction

Assessment of biological and health effects of low dose and low dose rate ionizing radiation (IR) exposure to human population is a thrust area of research as it provides important information for radiation protection science and risk estimation on human health. Delineating the effect of low dose radiation (LDR) in human population in the presence of several confounding, competing and risk modifying factors is a challenging task. Risk due to LDR is estimated from scenarios in which people are exposed to elevated level of natural background radiation, occupational exposure in nuclear establishments, diagnostic medical exposures, accidental exposures during and/or after nuclear disasters such as Chernobyl, Fukushima etc., and incidental exposures. The residents of high level natural radiation areas (HLNRAs) are exposed to chronic LDR at all developmental stages from birth to death and thus, provide unique opportunity to investigate biological effect of LDR directly in humans. The prominent HLNRAs in the world are Kerala (India), Yangjiang (China), Ramsar (Iran) and Guarapari (Brazil), where the level of natural background radiation is much higher (sometimes 10–100 times) as compared to adjacent normal level natural radiation areas (NLNRA) which are considered as control areas. The HLNRA of Kerala coast is known for its high population density and wide range of radiation dose levels ranging from < 1.0mGy to 45.0mGy/year due to the patchy distribution of monazite in the beach sand [1].

Risk assessment of health effects such as of cancer and hereditary diseases/disorders due to LDR exposure is estimated by extrapolation from the risk observed at high acute dose exposures such as data of atomic bomb survivors in Hiroshima and Nagasaki, assuming Linear No Threshold (LNT) hypothesis [2, 3]. The LNT hypothesis is used for regulatory and safety purposes, which assumes that every incremental small dose can lead to adverse health effects. However, it is highly debated for the lack of scientific validity or evidence. Epidemiological studies require large sample size for statistical validity to estimate the direct risk from LDR exposures. At the same time, biological mechanisms such as adaptive response, bystander effect, genomic instability and abscopal effects [4] etc. and inter-individual variation in terms of radio-sensitivity, make the issue of LDR risk estimation even more complex, as the available information is limited.

In recent years, one of the prime focuses in radiation protection science is to understand biological mechanisms in response to LDR [5]. Quantification of DNA damage including DNA double strand breaks (DSBs), its repair efficiency, somatic and germinal mutation rates etc., are of high relevance and may have tremendous implications towards validating LNT hypothesis, as there are limitations regarding experimental data to support the mechanistic effect of LDR. Risk assessment due to LDR exposure on human health is also highly relevant as there is increasing use of radiation in health care system for medical diagnosis, interventional radiology and radiotherapy of cancer. Experimental data at low doses is insufficient as compared to high dose range, where plenty of data is available for epidemiology. Considerable progress has been made in assessing the dose response relationship of cancer and non-cancer diseases/disorders as well as biological response at low dose exposures by generating data on computed tomography (CT) scanning [6–8], natural background radiation [9–14] and other radiation exposed population based studies [15, 16]. With the advent of newly discovered high throughput techniques, the underlying biological mechanisms are being explored by using sensitive assays and suitable bio-markers for assessment of cellular and molecular responses at low doses below 100 mGy.

IR induces different types of DNA lesions including DSBs that can be lethal to cells. It may lead to accumulation of mutations, cell death and carcinogenesis, if not repaired and/or mis-repaired. Conventional techniques such as chromosomal aberration analysis, micronuclei, premature chromosome condensation, pulsed field electrophoresis and comet assays are used to detect DNA damage in cells exposed to IR [17]. γH2AX assay is considered as one of the most sensitive methods to measure DNA damage such as DNA DSBs for radiation exposure. γH2AX is the phosphorylated form of the histone H2 variant H2AX and accumulates several DNA damage response (DDR) proteins such ATM, DNAPKcs, 53BP1, RAD50 etc., at the site of DNA damage [18, 19] forming IR induced foci (IRIF) [20, 21]. It is also reported that number of γH2AX foci is highly correlated with DNA DSBs [22]. The baseline frequency of DNA lesions, i.e., single strand breaks, DSBs, base damages etc., induced by IR is less as compared to the DNA damage produced endogenously [23]. Several studies are conducted using DDR proteins such as ATM and 53BP1 as biomarkers along with γH2AX using immunofluorescence by microscopy or flow cytometry. The γH2AX foci have been used as a biomarker of DSBs in human population exposed to natural background radiation, occupational, diagnostic and radio-therapeutic exposures and also radiation triage or accidently exposed populations [18, 24–34].

Extrapolation of risk from at high doses to low doses assumes that stochastic health effects and DNA damage at high and low doses are similar and the dose response is linear. It has been found that the induction of DNA DSBs may not always show a linear dose response due to IR exposure [35–38]. Although at high doses of IR exposure biological end points show linear dose response, but at low doses, it is not always linear. LNT hypothesis has been challenged scientifically as in vitro and in vivo studies have demonstrated inconsistent biological responses, particularly non-linear dose-responses and beneficial effects due to low dose radiation [39–41]. Accumulating evidences reveal that low and high doses have different biological responses and effects. Low doses of IR also induce lesser DNA damage and show efficient DNA repair [22, 29, 30]. Hence, it is ideal to conduct experiments using sensitive DNA damage markers to study the shape of the dose response curve in human populations exposed in vivo to low dose and low rate IR exposures. Several studies have been conducted in HLNRA of Kerala coast by employing phenotypic, cytogenetic and DNA damage parameters and no dose related increase was observed in any of the end points studied [13–14, 29–30; 42–55]. Hence, further studies on dose response relationship are essential using sensitive markers in different dose groups of HLNRA in Kerala coast.

In the present study, the basal level frequency of DNA DSBs in vivo was estimated in peripheral blood mononuclear cells (PBMCs) using highly sensitive γH2AX as marker among the individuals from NLNRA and different dose groups of HLNRAs of Kerala coast. Attempt has been made to delineate the shape of the dose response curve at low dose and low dose rates using DNA DSB as an endpoint. Additionally, influence of age and personal habits such as smoking and alcoholism was evaluated on DNA DSBs from these individuals.

## Materials and methods

### Sample collection and ethics statement

Venous blood samples (2 ml) were collected from 200 random healthy male individuals from NLNRA (N = 50) and from four different dose groups of HLNRAs (N = 150) of Kerala coast. All the blood samples were collected with written informed consent, which was approved by the Medical Ethics Committee, Bhabha Atomic Research Centre, Trombay, Mumbai, India. A detailed questionnaire was used to obtain information on confounding factors such as age, habits like smoking and drinking alcohol. For γH2AX foci analysis all 200 male individuals were analysed (78 smokers and 144 individuals consume alcohol). For co-localization study of γH2AX and 53BP1, six healthy male individuals analysed (NLNRA, n = 3; HLNRA, n = 3), were non- smokers, non-drinkers and without having any chronic illness.

### Dosimetry

External gamma radiation levels were measured inside and outside of each house at one-meter height using a halogen quenched Geiger Muller (GM) tube-based survey meter (Type ER-709, Nucleonix Systems, India). The survey meter readings measured absorbed doses in air (µR/h), which were converted to annual dose (mGy/year) using a conversion factor of 0.0765 (= 0.873 × 24 h × 365 days × 10^− 5^). Individual dose was calculated by multiplying inside and outside doses with the occupancy factor of 50:50 [10]. The radiation dose of ≤ 1.5 mGy/year (n = 50) was considered as NLNRA and > 1.50 mGy/year, as HLNRA (n = 150). HLNRA samples were further split into four dose groups of 1.51-5.0 mGy/year (n = 50), 5.01-10.0 mGy/year (n = 30), 10.01–15.0 mGy/year (n = 33), and > 15.0 mGy/year (n = 37). Co-localization of γH2AX and 53BP1 markers were analysed in 3 individuals from NLNRA (Mean dose: 1.34 ± 0.13 mGy/year) and 3 individuals from HLNRA (Mean dose: 33.48 ± 5.82 mGy/year).

### Isolation of PBMCs from human blood

PBMCs were separated from the venous blood samples by density gradient centrifugation using Histopaque-1077 solution (Sigma Aldrich, St. Louis, MO, USA). Equal volume of blood was overlaid on Histopaque solution and centrifuged at 400 g for 30 min at room temperature. Interface opaque layer containing PBMCs was carefully aspirated and washed with chilled isotonic phosphate-buffered saline (PBS) twice and processed further.

### Immunofluorescence staining using γH2AX and 53BP1 as biomarkers

All the samples were processed for γH2AX, while a subset of six samples (NLNRA, n = 3; HLNRA, n = 3) were analysed for co-localization of 53BP1 foci with γH2AX. Sample preparation for immunofluorescence staining was done as per the protocol described elsewhere (Jain et al., 2016). Briefly, PBMCs were fixed with freshly prepared chilled 1% formaldehyde (Sigma Aldrich) on ice for 15 min. After fixation, cells were washed with PBS (pH 7.4) and re-suspended in freshly prepared 70% ethanol and stored at -20°C until further processing. The PBMCs were permeabilized with 0.2% Triton-X-100 solution (Sigma Aldrich, USA) for 5 min at room temperature followed by blocking with 1% bovine serum albumin (Sigma Aldrich, USA) and incubated for overnight at 4°C in 1:100 (10 g/ml) concentration of anti-phospho-histone H2AX (Ser139), antibody (Upstate-Millipore 05-636, CA, USA) and Alexa Fluor 546 anti-rabbit antibody for 53BP1 (Molecular probes, USA). Cells were then washed in 1% blocking solution and labelled with Alexafluor-488 conjugated rabbit anti-mouse antibody (Molecular probes A-11,059, Eugene, USA) for 1 h at room temperature. Cells were washed with PBS and layered onto poly-l-lysine coated coverslips (BD Bio Coat 354,085, USA) and kept for 30 min at room temperature for adherence and mounted onto glass slides using prolong gold antifade DAPI reagent (Molecular Probes P 36,931, USA).

### Co-localization of γH2AX and 53BP1 markers

Co-localization of 53BP1 foci with γH2AX foci was analysed in a subset of six samples [NLNRA (n = 3) and HLNRA (n = 3)]. After fixation and permeabilization, cells were co-incubated in mouse monoclonal γH2AX and rabbit monoclonal 53BP1 antibodies overnight. Secondary antibody incubation was done using Alexa Fluor 488 conjugated anti-mouse (Molecular probes, USA) for γH2AX and Alexa Fluor 546 anti-rabbit antibody for 53BP1 (Molecular probes, USA). Frequency of γH2AX, 53BP1 and co-localized foci for both the markers were measured.

### Analysis of γH2AX and 53BP1 foci

The slides were examined at 40**×** magnification using fluorescence microscope (Carl Zeiss Microscopy GmbH, Germany). All the slides were blind-coded and around 20–25 random images with well-spread independent cells were captured for each sample. Around 250–300 cells were scored for γH2AX foci for each individual in semi-automated manner from captured images. The number of γH2AX foci in each cell and cells with one or more foci such as cells with 1, 2, 3, 4 or 5 foci were recorded. Scoring of γH2AX foci was performed by three independent scientists.

### Statistical analysis

The distribution of γH2AX foci per cell (total number of γH2AX foci ÷ total no. of cells scored) was approximately normal and hence independent t-test was used for comparison between two groups and ANOVA for comparison among more than two groups. Linear regression was employed to explore the relationship between residential dose, age of the donor, smoking and drinking status vis-a-vis the number of γH2AX foci per cell. Box and whisker plot was used for depicting the distribution γH2AX foci per cell in different subgroups. Chi-square test was employed to compare the distribution of cells with 1, 2, 3, 4 and 5 foci across different groups. STATISTICA software (version 9.1) was used for the statistical analysis. Significance level was kept at 5% and no adjustments were carried out for multiple comparisons.

## Results

In the present study, DSBs were measured using γH2AX as a marker. The frequencies of DSBs were measured as foci counts in each cell, where each of the foci was interpreted as a DSB. DNA DSBs were quantitated among 200 healthy male individuals (15 to 59 years) with a mean age of 38.1 ± 8.3 years. Fifty individuals were from NLNRA and 150 were from HLNRA dose groups. The mean age of the individuals from NLNRA and HLNRA dose groups was 39.2 ± 8.4 years and 37.7 ± 8.2 years, respectively.

**Fig. 1:**
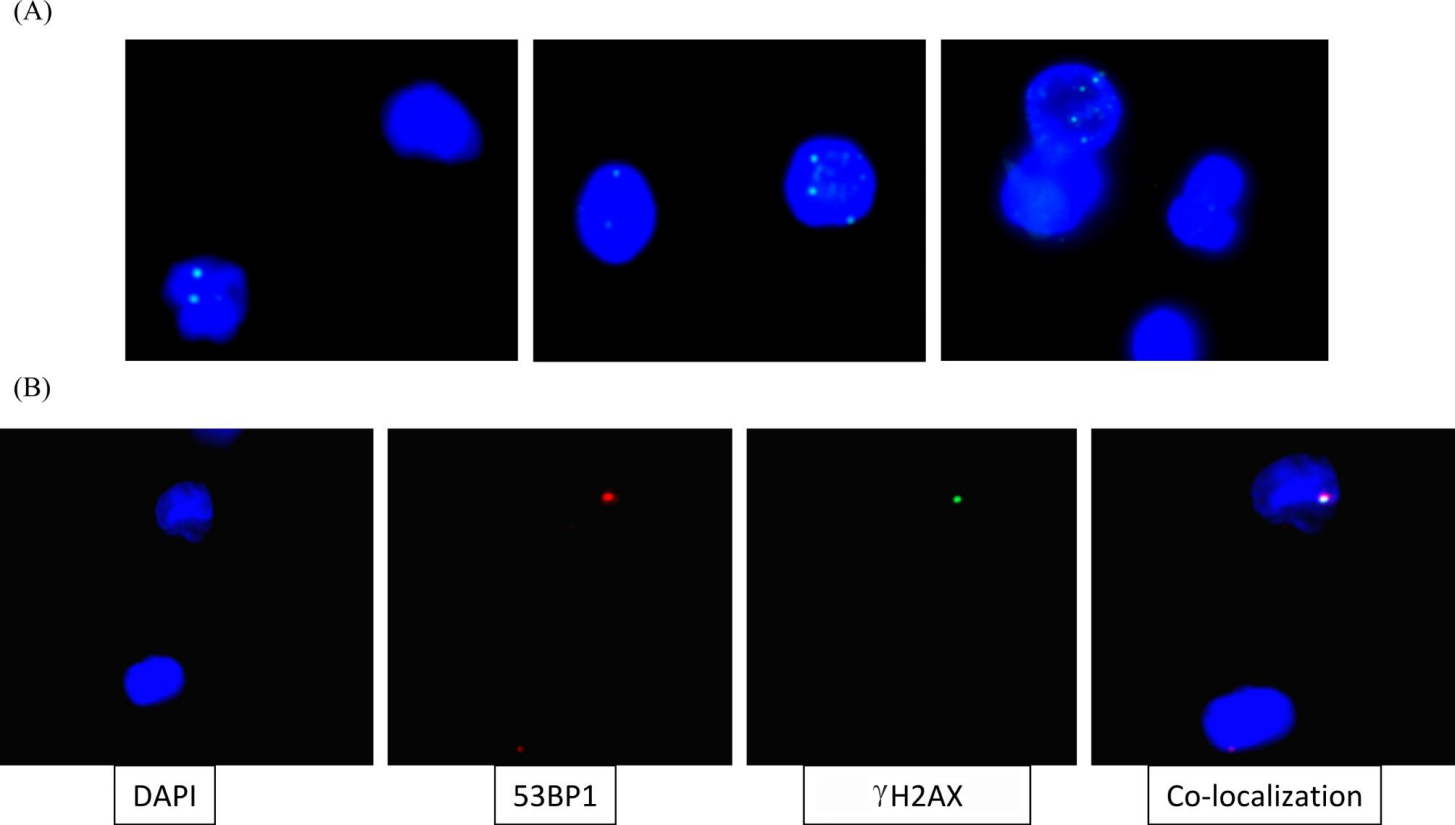
Representative image showing (A) distribution of one or multiple γH2AX foci per cell (B) Co-localization of γH2AX and 53 BP1 foci in peripheral blood mononuclear cells

**Fig. 2:**
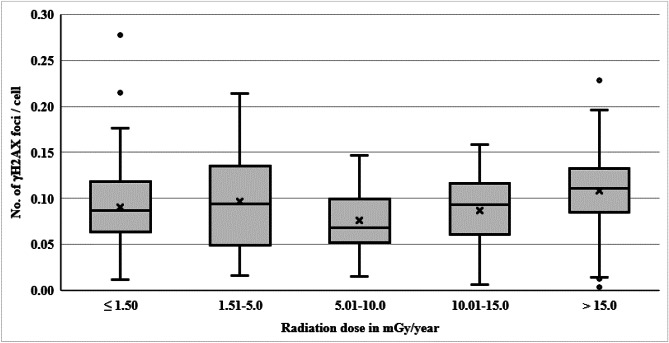
Box plot showing distribution of basal level frequency of γH2AX foci / cell in peripheral blood mononuclear cells of individuals (n = 200) from different back-ground dose groups. Dose group ≤ 1.50mGy/year is considered as NLNRA (control group). Dose groups such as 1.51-5.0mGy/year, 5.01 -10.0mGy/year, 10.01-15.00mGy/year, and > 15mGy/year are considered as HLNRA (exposed group). NLNRA: Normal level Natural radiation areas; HLNRA: High level natural radiation area

The γH2AX foci were scored from 52,223 cells with an average of 261 cells per individual (range: 109 to 390 cells/individual) and γH2AX foci were observed in 4,164 (7.97%) cells. The number of cells with 1, 2, 3, 4 and 5 foci were 3,719 (7.12%), 349 (0.67%), 74 (0.14%), 15 (0.03%) and 7 (0.01%), respectively. The mean frequency of γH2AX was 0.092 ± 0.047 foci per cell (range: 0.003–0.28) and a maximum number of foci per cell observed was five (Table 1). A representative image of cells with multiple foci is shown in Fig. 1A. The mean dose of fifty individuals from NLNRA was observed to be 1.21 ± 0.21 mGy/year (ranged from 0.57 to1.49 mGy/year) and that of 150 individuals from HLNRA was 10.52 ± 7.93 mGy/year (ranged from 1.57 to 39.88 mGy/year). The frequency of γH2AX foci per cell was similar among individuals from NLNRA and HLNRA population (0.090 ± 0.051v/s 0.096 ± 0.051; t_198_ = 0.33; P = 0.739). As shown in Table 1; Fig. 2, the frequency of γH2AX foci / cell in different dose groups of HLNRA was observed to be 0.096 ± 0.051, 0.076 ± 0.036, 0.087 ± 0.042, and 0.108 ± 0.046, among individuals with radiation doses of 1.51-5.0 mGy/year (mean dose : 3.02 ± 0.95; range: 1.57–4.93), 5.01 -10.0 mGy/year (mean dose : 7.43 ± 1.48; range: 5.01–9.75), 10.01-15.0 mGy/year (mean dose : 12.22 ± 1.47; range: 10.21–14.99), > 15.0 mGy/year (mean dose: 21.64 ± 6.28; range: range: 15.26–39.88), respectively and was statistically similar to NLNRA (F_4,195_ = 2.18, P = 0.072).

**Table 1:** Distribution of mean γH2AX foci/cell ± SD in PBMCs of individuals from NLNRA and different dose groups of HLNRA

Area (Dose in mGy/year)	n	Age (years ± SD)	Mean Dose (mGy/y ± SD) and Range	Total no. of Cells scored (no. of γH2AX foci observed)	Mean γH2AX foci/ cell ± SD	Number of cells with				
						1 γH2AX focus (%)	2 γH2AX foci (%)	3 γH2AX foci (%)	4 γH2AX foci (%)	5 γH2AX foci (%)
NLNRA (≤ 1.50)	50	39.2 ± 8.4	1.21 ± 0.21 (0.57–1.49)	13,221 (1181)	0.090 ± 0.051	990 (7.49)	68 (0.51)	17 (0.13)	1 (0.01)	0 (0)
HLNRA (> 1.50)	150	37.7 ± 8.2	10.52 ± 7.93 1.57–39.88	39,002 (3553)	0.096 ± 0.051	2729 (7.00)	281 (0.72)	57 (0.15)	14 (0.04)	7 (0.02)
NLNRA + HLNRA	200	38.1 ± 8.3	8.19 ± 7.96 (0.57–39.88)	52,223 (4734)	0.092 ± 0.047	3719 (7.12)	349 (0.67)	74 (0.14)	15 (0.03)	7 (0.01)
HLNRA dose groups (mGy/year)										
1.51-5.0	50	35.9 ± 8.5	3.02 ± 0.95 (1.57–4.93)	13,055 (1225)	0.096 ± 0.051	913 (6.99)	107 (0.82)	15 (0.11)	7 (0.05)	5 (0.04)
5.01-10.0	30	35.7 ± 8.1	7.43 ± 1.48 (5.01–9.75)	8169 (617)	0.076 ± 0.036	504 (6.17)	44 (0.54)	7 (0.09)	1 (0.01)	0 (0)
10.01-15.0	33	38.6 ± 7.5	12.22 ± 1.47 (10.21–14.99)	8637 (746)	0.087 ± 0.042	578 (6.69)	55 (0.64)	15 (0.17)	2 (0.02)	1 (0.01)
> 15.0	37	40.9 ± 7.9	21.64 ± 6.28 (15.26–39.88)	9141 (965)	0.108 ± 0.046	734 (8.03)	75 (0.82)	20 (0.22)	4 (0.04)	1 (0.01)

Comparison of γH2AX frequency between NLNRA and HLNRA: t_198_ = 0.33; P = 0.739. SD: Standard Deviation

Comparison of γH2AX frequency across NLNRA and different residential groups in HLNRA: F_4,195_ = 2.18; P = 0.072

NLNRA: Normal level natural radiation area; HLNRA: High level natural radiation area; PBMCs: Peripheral Blood mononuclear cells

We observed similar mean frequencies of γH2AX foci (0.10 ± 0.083) and 53BP1 (0.108 ± 0.083) in a NLNRA [n = 3, mean dose: 1.34 ± 0.13 mGy/year, mean age: 42.0 ± 2.0 years) and HLNRA (n = 3, mean dose: 33.48 ± 5.82 mGy/year, mean age: 45.0 ± 2.5 years] which showed good correlation between both the DSB markers. Representative images showing co-localisation of γH2AX and 53 BP1 marker is shown in Fig. 1B.

As shown in Fig. 2, there is an apparent non-linear dose-response between DNA DSBs in terms of γH2AX foci / cell and background radiation. There is a decrease in γH2AX foci/cell, though not statistically significant, in the dose group of 5.01 to 10mGy/year (Table 1; Fig. 2). As depicted in supplementary Fig. 1, linear-quadratic equation fits the relationship between γH2AX foci/cell and background radiation in mGy/year better (R^2^ = 6.92%, P = 0.0009) with statistically significant linear (P = 0.0322) as well as quadratic (P = 0.0018) coefficients compared to linear equation (R^2^ = 2.21%, P = 0.0357).

To assess the effect of age on DNA DSBs in terms of γH2AX foci, the samples were stratified into six age groups i.e., ≤ 29, 30–34, 35–39, 40–44, 45–49 and ≥ 50 years. The frequency of γH2AX foci per cell was observed to be 0.098 ± 0.042, 0.078 ± 0.037, 0.084 ± 0.042, 0.099 ± 0.058, 0.097 ± 0.06 and 0.114 ± 0.033, respectively (Table 2). ANOVA suggested that the variation in the frequencies γH2AX foci were within the limits of random fluctuation (F_5,194_ = 2.18, P = 0.06). As given in Table 2, the frequency of DNA DSBs in terms of γH2AX foci did not show any significant difference between non-smokers and smokers (0.092 ± 0.047v/s 0.093 ± 0.048, t_198_ = 0.13; P = 0.895) and those who do and do not consume alcohol (0.091 ± 0.045 v/s 0.096 ± 0.052, t_198_ = 0.62; P = 0.538). Distribution of cells with 0, 1, 2, 3, 4 and 5 foci cells with respect to radiation dose, age, smoking and drinking habits depicted in Tables 1 and 2, which do not seem to suggest any significant difference.

**Table 2:** Distribution of mean γH2AX foci/cell ± SD according to age of the donor and personal habits

Characteristics	n	Mean Age in years ± SD	Mean dose in mGy/y ± SD	Total no. of Cells scored (no. of γH2AX foci observed)	Mean γH2AX foci/ cell ± SD	Number of cells with				
						1 γH2AX focus (%)	2 γH2AX foci (%)	3 γH2AX foci (%)	4 γH2AX foci (%)	5 γH2AX foci (%)
Age years ≤ 29	31	26.0 ± 3.6	6.37 ± 5.53	8366 (789)	0.098 ± 0.042	578 (6.91)	79 (0.94)	13 (0.16)	1 (0.01)	2 (0.02)
30–34	39	31.7 ± 1.4	6.87 ± 5.97	10,182 (790)	0.078 ± 0.037	647 (6.35)	47 (0.46)	12 (0.12)	2 (0.02)	1 (0.01)
35–39	45	36.9 ± 1.5	7.08 ± 6.91	11,838 (977)	0.084 ± 0.042	787 (6.65)	63 (0.53)	11 (0.09)	4 (0.03)	3 (0.03)
40–44	36	42.0 ± 1.5	9.22 ± 9.08	9241 (892)	0.099 ± 0.058	730 (7.9)	59 (0.64)	12 (0.13)	2 (0.02)	0 (0)
45–49	29	46.8 ± 1.3	12.65 ± 9.73	7410 (694)	0.097 ± 0.06	541 (7.3)	47 (0.63)	14 (0.19)	3 (0.04)	1 (0.01)
≥ 50	20	52.0 ± 2.8	7.76 ± 9.87	5186 (592)	0.114 ± 0.033	436 (8.41)	54 (1.04)	12 (0.23)	3 (0.06)	0 (0)
Smoking - No	122	37.5 ± 8.6	7.8 ± 7.35	32,256 (2911)	0.092 ± 0.047	2288 (7.09)	213 (0.66)	41 (0.13)	11 (0.03)	6 (0.02)
Smoking - Yes	78	38.9 ± 7.7	8.8 ± 8.85	19,967 (1823)	0.093 ± 0.048	1431 (7.17)	136 (0.68)	33 (0.17)	4 (0.02)	1 (0.01)
Drinking No	56	38.2 ± 9.4	7.77 ± 8.6	15,124 (1403)	0.096 ± 0.052	1062 (7.02)	109 (0.72)	25 (0.17)	7 (0.05)	4 (0.03)
Drinking - Yes	144	38.0 ± 7.9	8.35 ± 7.72	37,099 (3331)	0.091 ± 0.045	2657 (7.16)	240 (0.65)	49 (0.13)	8 (0.02)	3 (0.01)
Total	200	38.1 ± 8.3	8.19 ± 7.96	52,223 (4734)	0.092 ± 0.047	3719 (7.12)	349 (0.67)	74 (0.14)	15 (0.03)	7 (0.01)

Comparison of γH2AX frequency across age groups: F_5,194_ = 2.17; P = 0.059;

Comparison of γH2AX frequency between smokers and non-smokers: t_198_ = 0.13; P = 0.895;

Comparison of γH2AX frequency between those who do and do not consume alcoholic drinks: t_198_ = 0.62; P = 0.538

**Fig. 3:**
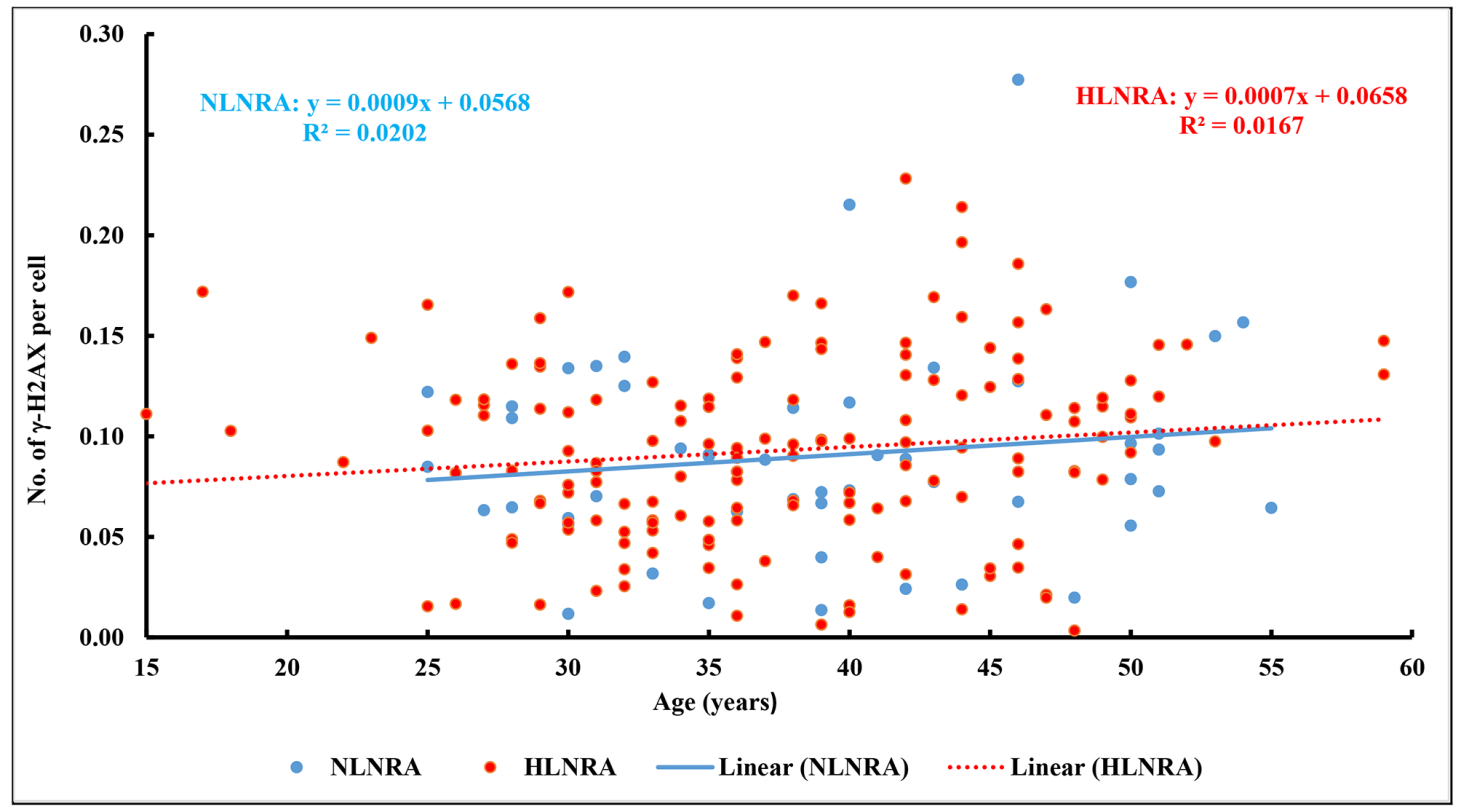
The frequency of γH2AX foci in different age groups of NLNRA and HLNRA individuals (n = 200). NLNRA: Normal level natural radiation area; HLNRA: High level natural radiation area

The relationship between age and frequency of γH2AX foci was explored further separately in individuals from NLNRA (regression coefficient: 0.0009 ± 0.0009, P = 0.325, R^2^ = 2.0%) and HLNRA (regression coefficient: 0.0007 ± 0.0005, P = 0.048, R^2^ = 1.7%) and does not seem to suggest any differential age effect among individuals from NLNRA and HLNRA (Fig. 3).

**Fig. 4:**
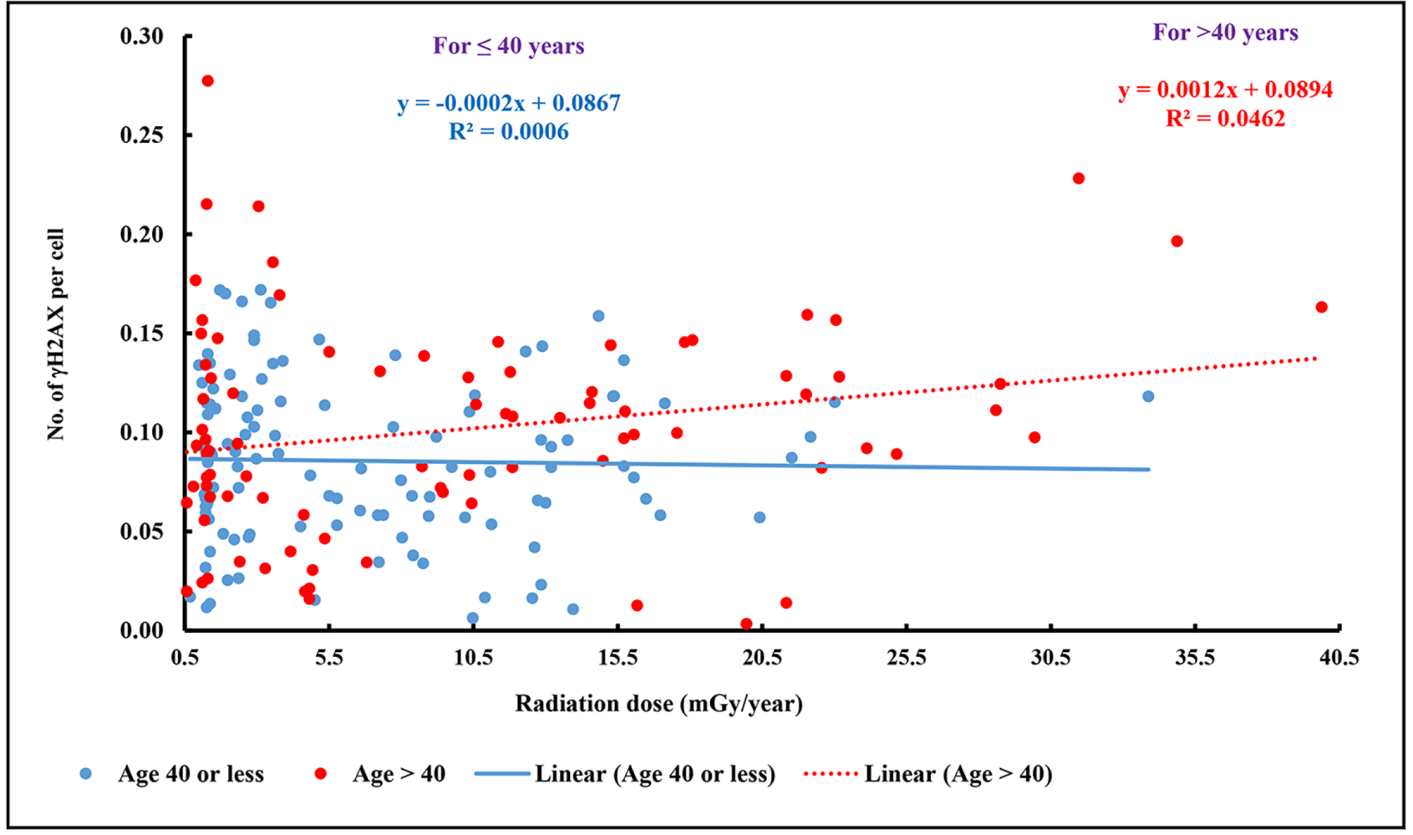
Relationship between radiation dose and γH2AX foci /cell in PBMCs of individuals in age groups ≤ 40 years and > 40 years (n = 200)

There was an apparent difference (Fig. 4) in the relationship between residential dose and γH2AX foci in individuals aged less than 40 years (regression coefficient: -0.0002 ± 0.0006, P = 0.792, R^2^ = 0.06%) compared to those aged 40 years or more (regression coefficient: 0.0012 ± 0.0006, P = 0.048, R^2^ = 4.6%), the interaction effect was not statistically significant (P = 0.119). As depicted in supplementary Fig. 2, the mean number of γH2AX foci per cell was higher among individuals belonging to > 40 years in all the residential dose groups except in those with 1.51-5.0 mGy/year. The mean number of γH2AX foci in NLNRA and HLNRA in different age groups shown in supplementary Fig. 3 do not seems to suggest any pattern, mean appears to be marginally higher in NLNRA among individuals aged 30–34 and 45–49 years and vice e versa in the other age groups of ≤ 29, 35–39, 40–44 and ≥ 50 years.

There was no evidence to suggest that habits such as smoking (P = 0.895) and drinking (P = 0.538) had any effect on the induction of DNA DSBs in terms of γH2AX foci. The linear-quadratic relationship between the foci/cell and background radiation remained unaltered even when a multiple linear regression analysis was carried out to assess the overall effect of radiation dose in mGy/year and age in years (as continuous variables) and; smoking and drinking as indicator variables. The regression equation was: Foci/cell = 0.081 − 0.002×dose + 0.0001×dose^2^ + 0.0005×age − 0.002×Smoking − 0.002×Drinking, R^2^ = 7.6%. Both the linear (P = 0.042) and quadratic (P = 0.004) regression coefficient of background radiation was statistically significant.

## Discussion

The present study is focused on the dose response relationship between DNA DSBs and background radiation dose in peripheral blood mono-nuclear cells of random healthy donors from HLNRA of Kerala coast. Mis-repaired or unrepaired DNA DSBs are highly deleterious and it may pose threat to cell viability, genome stability of the cell and the integrity of the genome. There are several techniques to measure DNA DSBs in human cells at G_0_ such as comet assay, pulsed field electrophoresis and cytogenetics to detect chromosomal aberrations like dicentric and translocation assay. In recent years, estimation of DNA DSBs using γH2AX marker has been extensively used to quantify radiation induced DNA DSBs for population monitoring, medical exposure, natural background, and occupational exposure situations [22, 29, 30, 42].

Dose response relationship at acute dose radiation exposures above 100 mGy mostly remains linear in human lymphocytes using chromosomal aberrations, micronuclei and γH2AX foci analysis [35–38]. However, the shape of the dose response curve at chronic low dose exposures below 100 mGy is found to be non-linear in human lymphocytes using various DNA damage endpoints [29, 43–45]. The present study evaluated dose response relationship between DSBs in terms of γH2AX foci and chronic low dose and low dose rate IR below 100mGy, ranging from 0.5 to 39.88 mGy/year with a larger sample size.

Studies have shown that the number of γH2AX foci is proportional to radiation dose from 1 mGy to 2 Gy for X rays [22, 46]. The frequency of DSB, estimated using γH2AX foci at background dose level of 10 mGy, was found to be ~ 0.3 DSB/cell. Studies by Rothkam and Lobrich (2003) reported the background γH2AX foci/cell to be 0.05 using primary human lung MRC-5 fibroblast cells [22]. Asaithamby and Chen (2009) used a live study with a tagged DNA damage marker (i.e., 53BP1-GFP) in immortalized human bronchial epithelial cells and did not observe any foci prior to IR exposure. The number of DSBs formed was linear with respect to radiation dose from 5 mGy to 1 Gy. The repair efficiency of DSBs induced by very low radiation doses (5 mGy) and by higher doses was reported to be similar [46]. Although the studies using cell lines at very low dose and higher doses reported linear relationship between DSBs and IR, enumeration of γH2AX foci in both the studies were done at different time after exposure to IR. Hence, dissimilarities such as cell types and methods of analysis for quantification of γH2AX foci have different implications [22, 46]. Another study showed an average of 21 radiation induced foci (RIF)/Gy between 0.05 and 0.25 Gy in 18 independent human fibroblast cell lines [47]. In contrast, Neumaier et al. (2011) using live imaging and mathematical fitting of RIF kinetics showed that RIF induction rate increased with increasing radiation dose, whereas the rate at which RIFs disappear decreased [48].

Radiation-induced γH2AX and 53BP1 nuclear foci are considered as useful markers for detecting radiation exposure at low doses below 20 mGy [18]. Risks associated with low-dose and low dose rate IR is important to understand the cellular responses to low doses of IR on human population. IR induces a plethora of DNA lesions including DNA double strand breaks and non-DSB clustered DNA damages in a cell. The amount of endogenous damage is high as compared to IR induced DNA damages. Among these, DNA DSBs are comparatively low and quantifiable per 1 Gy of low LET radiation [23].

Spontaneous DSB/foci levels are found to be much lower for non-cycling cells such as quiescent lymphocytes [42]. Due to diminishing signal to noise ratio and lack of other more sensitive techniques, γH2AX foci analysis needs larger sample size to establish conclusive results at lower doses to the tune of few mGy [42]. The present study analysed basal level γH2AX foci in relatively large sample size of 200 individuals, scoring as many as 52,223 cells at G_0_ from NLNRA and HLNRA individuals. Advantages of G_0_ PBMCs is that it avoids the proliferation factor, which may have influenced the foci formation. The basal level γH2AX foci were counted in each individual by taking an average of 261 cells (ranging between 109 and 390 cells). Cells with 0, 1 and multiple γH2AX foci were scored and their importance was highlighted. Very few cells were observed with large foci size which could be due to clustered lesions or fusion of two or more foci or RIFs or foci repair centres.

The present study includes a large sample size (n = 200) and five background dose groups with a sample size > 30 from Kerala coast. Approximately 6–7% cells have one focus, whereas ~ 1% cells had multiple foci indicating that damages to cells were similar in all the background dose groups studied. Above 92% cells did not contain γH2AX foci throughout the dose groups suggesting that induction and repair of DSBs with respect to chronic low dose from < 1.0 mGy to > 20 mGy showed similar response. Even ~ 20 times higher background dose in HLNRA as compared to NLNRA do not seem to induce statistically significant increase in the number of DSBs in terms of γH2AX foci in the present study. Interestingly, nonlinear trend of DSBs was observed in different background dose groups which is an important finding related to risk estimation of DSBs in response to chronic low dose IR. This data supports other epidemiological and biological studies conducted in this area in newborns and adults [13–[14, 29]–30, 49–59, 60], where we have not observed any increased dose response at any of the end points studied.

The frequency of γH2AX foci has shown a marginal reduction of DSBs at dose groups of 5.01 to 10.0 mGy/year and 10.01-15.0mGy/year. The reason at this stage is not clear but the observation might be suggestive of a threshold dose for this particular biological end-point. Our earlier studies on DNA damage and transcriptome analysis have shown similar findings, where above 5.0 mGy/year, a reduction of damage and abundance of DDR and repair genes observed in HLNRA (> 5 mGy/year) [29, 30, 59, 61].

In conclusion, the present study revealed that chronic low dose and low rates of IR prevailing in Kerala coast did not increase the frequency of DSBs in human PBMCs. However, DNA damage analysis in higher dose groups is required to draw firm conclusions on dose response relationship between DSBs and radiation dose below 100mGy. Further research on higher background dose groups in HLNRA are required using high throughput genomic and epi-genomics studies to understand underlying biological mechanisms due to LDR. Additionally, DNA damage response, and histone/chromatin modification studies might throw some new insights about the induction of foci due to low doses of IR.

## Electronic supplementary material

Below is the link to the electronic supplementary material.

Supplementary Material 1

Supplementary Material 2

Supplementary Material 3

Supplementary Material 4

## Data Availability

Data will be available on reasonable request to corresponding author.
