# Supplementary material for: Non-linear dose response of DNA double strand breaks in response to chronic low dose radiation in individuals from high level natural radiation areas of Kerala coast
Source: Genes Environ. 2023 May 1;45:16. doi: 10.1186/s41021-023-00273-6 (PMC10150514; doi:10.1186/s41021-023-00273-6)
Supplement: Supplementary file 1 — Supplementary Material 1 [file 41021_2023_273_MOESM1_ESM.docx]

**Legends to Figures:**

**Figure 1** Figure 1 Representative image showing (A) multiple γH_2_AX foci per cell (B) Co-localization of γH_2_AX and 53 BP1 foci in peripheral blood mononuclear cells of an individual.

**Figure 2** Box plot showing distribution of basal level frequency of γH_2_AX foci / cell in peripheral blood mononuclear cells of individuals (n=200) from different back-ground dose groups. Dose group ≤1.50mGy/year is considered as NLNRA (control group). Dose groups such as 1.51-5.0mGy/year, 5.01 -10.0mGy/year, 10.01-15.00mGy/year, and >15mGy/year are considered as HLNRA (exposed group). NLNRA: Normal level Natural radiation areas; HLNRA: High level natural radiation area.

**Figure 3** The frequency of γH2AX foci in different age groups of NLNRA and HLNRA individuals (n=200). NLNRA: Normal level natural radiation area; HLNRA: High level natural radiation area.

**Figure 4** Relationship between radiation dose and γH2AX foci /cell in PBMCs of individuals in age groups ≤ 40 years and > 40 years (n = 200)

**Supplementary Figure 1** Distribution of γH_2_AX foci/cell in peripheral blood mononuclear cells (PBMCs) of individuals (n=200) from different background radiation dose levels

**Supplementary Figure 2** Box plot showing the distribution of ℽH2AX foci among individuals aged ≤40 and >40 years in six different radiation dose groups (≤1.5, 1.51-5.0, 5.01-10.0, 10.01-15.0, and > 15.0 mGy/year).

**Supplementary Figure 3** Box plots showing the distribution of ℽH2AX foci among individuals from age groups ≤40 and >40 years from NLNRA and HLNRA
