# Supplementary material for: Non-linear dose response of DNA double strand breaks in response to chronic low dose radiation in individuals from high level natural radiation areas of Kerala coast
Source: Genes Environ. 2023 May 1;45:16. doi: 10.1186/s41021-023-00273-6 (PMC10150514; doi:10.1186/s41021-023-00273-6)
Supplement: Supplementary file 2 — Supplementary Material 2 [file 41021_2023_273_MOESM2_ESM.docx]

Supplementary Figure 1 Distribution of γH_2_AX foci/cell in peripheral blood mononuclear cells (PBMCs) of individuals (n=200) from different background radiation dose levels
