# Supplementary material for: Non-linear dose response of DNA double strand breaks in response to chronic low dose radiation in individuals from high level natural radiation areas of Kerala coast
Source: Genes Environ. 2023 May 1;45:16. doi: 10.1186/s41021-023-00273-6 (PMC10150514; doi:10.1186/s41021-023-00273-6)
Supplement: Supplementary file 3 — Supplementary Material 3 [file 41021_2023_273_MOESM3_ESM.docx]

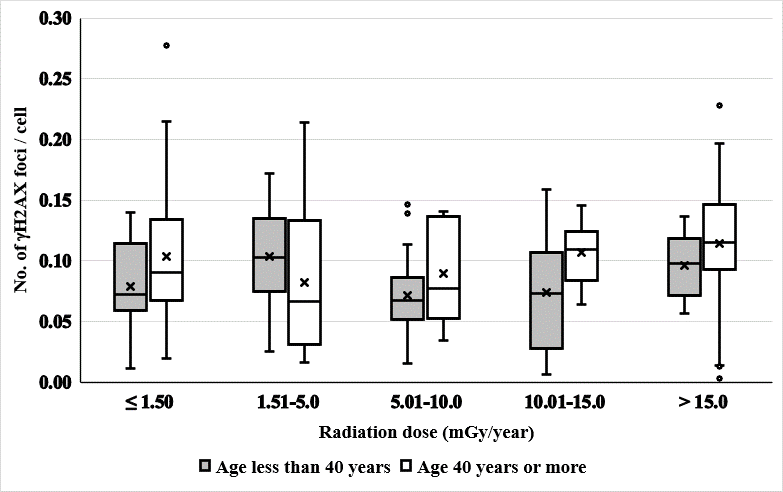


Supplementary Figure 2 Box plot showing the distribution of ℽH2AX foci among individuals aged < 40 years and ≥40 years in five different radiation dose groups (≤1.5, 1.51-5.0, 5.01-10.0, 10.01-15.0, and >15.0 mGy/year).
