# Supplementary material for: Non-linear dose response of DNA double strand breaks in response to chronic low dose radiation in individuals from high level natural radiation areas of Kerala coast
Source: Genes Environ. 2023 May 1;45:16. doi: 10.1186/s41021-023-00273-6 (PMC10150514; doi:10.1186/s41021-023-00273-6)
Supplement: Supplementary file 4 — Supplementary Material 4 [file 41021_2023_273_MOESM4_ESM.docx]

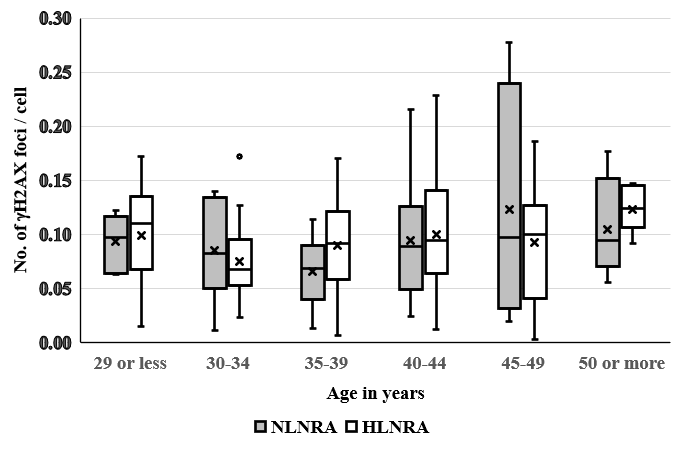


Supplementary Figure 3. Box plots showing the distribution of ℽH2AX foci among individuals from age groups < 40 years

and ≥40 years from NLNRA and HLNRA
